# Global Burden of Skin Disease Representation in the Literature: Bibliometric Analysis

**DOI:** 10.2196/29282

**Published:** 2021-08-31

**Authors:** Kayd J Pulsipher, Mindy D Szeto, Chandler W Rundle, Colby L Presley, Melissa R Laughter, Robert P Dellavalle

**Affiliations:** 1 College of Osteopathic Medicine Rocky Vista University Parker, CO United States; 2 Department of Dermatology University of Colorado Anschutz Medical Campus Aurora, CO United States; 3 Department of Medicine St. Joseph's Hospital Denver, CO United States; 4 Division of Dermatology Lehigh Valley Health Network Allentown, PA United States; 5 Department of Epidemiology Colorado School of Public Health University of Colorado Anschutz Medical Campus Aurora, CO United States; 6 Dermatology Service Rocky Mountain Regional Medical Center US Department of Veteran Affairs Aurora, CO United States

**Keywords:** global burden of disease, global health, global dermatology, disability-adjusted life years, GBD, DALYs, journalology, dermatology, skin disorders

## Abstract

**Background:**

The global burden of skin disease may be reduced through research efforts focused on skin diseases with the highest reported disability-adjusted life years.

**Objective:**

This study evaluates the representation of dermatologic conditions comprising the highest disability-adjusted life years in dermatology literature to identify areas that could benefit from greater research focus.

**Methods:**

The top 10 skin disorders according to their respective disability-adjusted life years as per the 2013 Global Burden of Disease were identified using previous studies. The top 5 dermatology journals ranked by the 2019 h-index were also identified. A PubMed search of each journal was performed using individual skin disease terms. From 2015 to 2020, all indexed publications pertaining to each disease were recorded and compared to the total number of publications for each journal surveyed.

**Results:**

A total of 19,727 papers were published in the 5 journals over the span of 2015-2020. Although melanoma ranked as the eighth highest in disability-adjusted life years, it had the highest representation in the literature (1995/19,727, 10.11%). Melanoma was followed in representation by psoriasis (1936/19,727, 9.81%) and dermatitis (1927/19,727, 9.77%). These 3 conditions comprised a total of 29.69% (5858/19,727) of the total publications, while the remaining 7 skin conditions were represented by a combined 6.79% (1341/19,727) of the total publications.

**Conclusions:**

This research identifies gaps in the literature related to the top skin diseases contributing to the global burden of disease. Our study provides insight into future opportunities of focused research on less-studied skin diseases to potentially aid in reducing the global burden of skin disease.

## Introduction

The 2013 Global Burden of Disease (GBD) Morbidity and Mortality report identified skin diseases as the fourth leading cause of global disability-adjusted life years (DALYs) [1]. One DALY is the sum of years of life lost to a disease plus years lived with disability, with 1 DALY equating to 1 year of healthy life lost [1]. Research pertaining to skin disorders with higher reported DALYs has potential to reduce the global burden of skin disease through improvements in management guidelines, public health initiatives, policy changes, and increased awareness within the scientific and greater community [2]. This study evaluates the representation of dermatologic conditions comprising the highest DALYs in dermatology literature to identify areas that could benefit from increased research focus.

## Methods

A comprehensive search was performed using PubMed to identify peer-reviewed papers. A previous GBD study has identified and ranked individual skin disorders according to their respective DALYs [1]. This GBD study was used to select our specific search terms such as dermatitis, acne vulgaris, psoriasis, urticaria, viral skin diseases, fungal skin diseases, scabies, melanoma, pyoderma, and cellulitis. The h-index is a noted metric used to measure individual author and journal research influence and impact [3]. The top 5 dermatology journals ranked by the 2019 h-index were identified using the Scimago Journal and Country Rank [4]. PubMed searching was performed by pairing individual skin disease terms with each journal title (eg, “dermatitis” AND “Journal of the American Academy of Dermatology”). From 2015 to 2020, all indexed publications pertaining to each disease were recorded and compared to the total number of publications for each journal surveyed. All article types were included to obtain a complete picture of relevant skin disease research. Duplicate papers were excluded.

## Results

**Figure 1:**
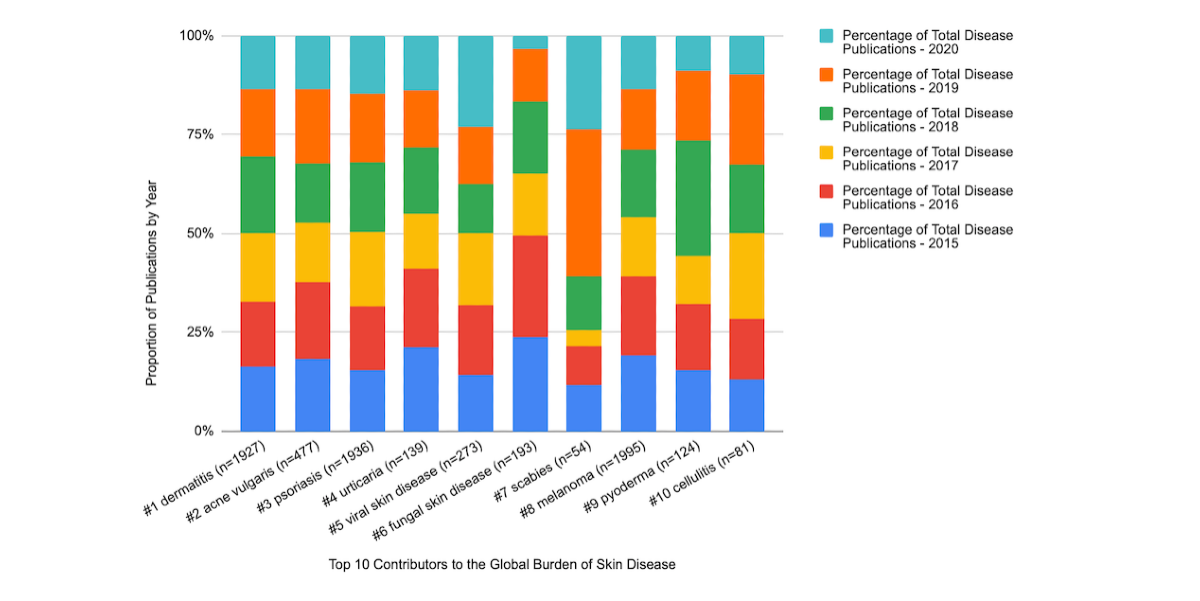
Proportion of publications by year for each global burden of skin disease condition.

Over the span of 2015-2020, 19,727 publications were recorded from the previously mentioned journals. Melanoma (eighth in DALYs) had the highest representation in the literature at 10.11% (1995/19,727) of the total publications, followed by psoriasis (1936/19,727, 9.81%) and dermatitis (1927/19,727, 9.77%) (Table 1). Melanoma, psoriasis, and dermatitis comprised a total of 29.69% (5858/19,727) of all the publications from 2015 to 2020. The remaining 7 skin diseases comprised only 6.79% (1341/19,727) of the total publications. Acne vulgaris, the second highest contributor to skin GBD, followed dermatitis with a much lower representation in the literature (477/19,727, 2.42%). Scabies accumulated the lowest percentage of the total publications (54/19,727, 0.27%). The proportions of publications by year for each disease are shown in Figure 1.

**Table 1:** Top 10 skin conditions contributing to the global burden of disease [1] and their representation in the dermatology literature.^a^

Skin disease search term	Global burden of skin disease rank	Rank by percentage of total publications	Percentage of global burden of disease (measured in disability-adjusted life years)^b^	Proportion of global burden of skin disease measured in disability-adjusted life years, fraction (%)^c^	Publications in 2015-2020 (N=19,727), n (%)	Percentage of total publications/Percentage of global burden of skin disease
Dermatitis	1	3	0.38	0.38/1.70 (22.35)	1927 (9.77)	0.44
Acne	2	4	0.29	0.29/1.70 (17.06)	477 (2.42)	0.14
Psoriasis	3	2	0.19	0.19/1.70 (11.18)	1936 (9.81)	0.88
Urticaria^d^	3	7	0.19	0.19/1.70 (11.18)	139 (0.70)	0.06
Viral skin disease	5	5	0.16	0.16/1.70 (9.41)	283 (1.38)	0.15
Fungal skin disease	6	6	0.15	0.15/1.70 (8.82)	193 (0.98)	0.11
Scabies	7	10	0.07	0.07/1.70 (4.12)	54 (0.27)	0.07
Melanoma	8	1	0.06	0.06/1.70 (3.53)	1995 (10.11)	2.86
Pyoderma	9	8	0.05	0.05/1.70 (2.94)	124 (0.63)	0.21
Cellulitis	10	9	0.04	0.04/1.70 (2.35)	81 (0.41)	0.17
All other skin and subcutaneous diseases	N/A^e^	N/A	0.12	0.12/1.70 (7.00)	N/A	N/A

^a^The following journals ranked by the 2019 h-index were searched: rank 1, Journal of the American Academy of Dermatology; rank 2, Journal of Investigative Dermatology; rank 3, British Journal of Dermatology; rank 4, Journal of the American Medical Association Dermatology; and rank 5, Dermatologic Surgery.

^b^The percentage values in this column have been directly taken from the global burden of disease paper [1]. Total skin-related percentage of global burden of disease=1.70%.

^c^This column shows the fractions of the total skin-related global burden of disease over the total percentage of global burden of disease (1.70%) calculated for the 10 skin diseases.

^d^Urticaria has the same ranking as psoriasis in the calculation of the global burden of skin disease rankings [1].

^e^N/A: not applicable.

## Discussion

The disproportionate representation of melanoma in the literature compared to overall GBD is likely explained by the increased mortality risk of melanoma relative to other skin diseases [5,6]. Additionally, a large portion of the examined publications originated from North American– and European-based journals, which are regions with high melanoma incidence reported globally [5]. Notably, these regions have minimal incidence of scabies [1], which had the lowest representation in the literature. However, literature representation is likely multifactorial, with epidemiologic factors and research funding contributing to literature representation [7].

Although this study utilizes 2013 GBD data to guide our literature search, it does not implicate the literature gaps identified in this study. Our study was limited by the use of 1 specific search term pertaining to the individual skin diseases. We recognize that performing our search across 5 journals with a single term per skin disease could have led to possible omissions. Although a variety of terms could be searched for some skin diseases within our study, such as fungal skin diseases, we elected to use a single term for consistency across all the skin diseases studied and recognize that certain publications discussing multiple skin diseases may have been listed under more than one search term. Lastly, we acknowledge that many of the mentioned skin diseases may be represented outside of dermatology-specific journals, which our study did not examine. Nonetheless, we offer a valuable initial survey of these skin diseases in highly read and influential dermatology literature and hope that our study will prompt future necessary work to identify potential avenues for refinement of current research efforts.

Indeed, a primary purpose of the GBD collaboration is to aid clinical researchers in determining priority of research at local, national, and global levels [1]. Herein, our study provides insight into possible future investigative pathways for dermatologic research. For example, urticaria accounts for 11.18% (0.19/1.70) of skin-related DALYs (equal to psoriasis), yet these rank sixth and second in the percentage of the total publications, respectively [8]. Thus, researchers have opportunities to further elucidate causal mechanisms and the clinical impact of less-studied dermatologic conditions as a means to guide clinical decision-making, public health initiatives, policy changes, and education for dermatologists.

Dermatologic disease is a significant source of global DALYs. Although there has been significant research focus on dermatologic malignancies, dermatitis, and psoriasis in the last 5 years, this study highlights significant gaps and opportunities that remain in skin disease literature.

## Abbreviations

DALY: disability-adjusted life year
GBD: Global Burden of Disease
